# Crystal structures of two solvates of (18-crown-6)potassium acetate

**DOI:** 10.1107/S2056989016017436

**Published:** 2016-11-08

**Authors:** Phil Liebing, Ahmad Zaeni, Falk Olbrich, Frank T. Edelmann

**Affiliations:** a Chemisches Institut der Otto-von-Guericke-Universität Magdeburg, Universitätsplatz 2, 39106 Magdeburg, Germany; b Chemistry Department, Haluoleo University, Kendari, Indonesia; c Institut für Anorganische und Angewandte Chemie der Universität Hamburg, Martin-Luther-King-Platz 6, 20146 Hamburg, Germany

**Keywords:** crystal structure, potassium, crown ether, 18-crown-6, acetate, hydrate, hydro­acetate, hydrogen bridge bonds

## Abstract

The crystal and mol­ecular strutures of two solvated forms of [K(18*c*6)]OAc (18*c*6 = 18-crown-6 = 1,4,7,10,13,16-hexa­oxa­cyclo­octa­decane and OAc = acetate) were determined by single-crystal X-ray diffraction, namely (acetato-κ^2^ *O*,*O*′)(1,4,7,10,13,16-hexa­oxa­cyclo­octa­decane-κ^6^ *O*)potassium dihydrate, [K(CH_3_COO)(C_12_H_24_O_6_)]·2H_2_O (**1**) and (acetato-κ^2^ *O*,*O*′)aqua­(1,4,7,10,13,16-hexa­oxa­cyclo­octa­decane-κ^6^ *O*)potassium acetic acid monosolvate [K(CH_3_COO)(C_12_H_24_O_6_)(H_2_O)]·CH_3_COOH (**2**). In both compounds, the acetate anion is bonded to the potassium ion in a chelating fashion and the metal atom is consequently slightly displaced from the O_6_ plane of the crown ether. In the crystals, O—H⋯O hydrogen bonds lead to a polymeric ladder structure in the dihydrate **1**, while the acetic acid hydrate **2** features inversion dimers.

## Abstract 2

Hydrogen bonding plays an important role in the structures of two solvated forms of [K(18*c*6)]OAc (18*c*6 = 18-crown-6 = 1,4,7,10,13,16-hexa­oxa­cyclo­octa­decane and OAc = acetate).

## Chemical context   

As a result of the macrocyclic ether 1,4,7,10,13,16-hexa­oxa­cyclo­octa­decane (‘18-crown-6’) being a hexa­dentate ligand that is highly specific for the potassium cation, it is frequently used to manipulate the properties of various potassium compounds. On the one hand, the [K(18c6)]^+^ cation (18c6 = 18-crown-6) is a powerful tool to crystallize large anions with the objective to make them accessible for single-crystal structure determination. Thus, the crystal structures of numerous anionic complex compounds have been observed from their [K(18c6)]^+^ salts, *e.g.* [HPMo_12_O_40_]^4–^ (Neier *et al.*, 1995 ▸) and [Hg*R* ^f^_2_ *X*]^−^ (*R* ^f^ = CF_3_, C_6_F_5_, *X* = Br, I; Schulz *et al.*, 2003 ▸) to mention just two examples among many. The same applies to a broad ensemble of unusual non-metal anions such as I_3_^−^ (Sievert *et al.*, 1996 ▸) and the radical species C_2_N_4_S_2_^−^ (Makarov *et al.*, 2005 ▸). Moreover, since the early days of crown-ether chemistry, 18-crown-6 has been used to enhance the solubility of reactive potassium salts in organic media, *e.g.* KMnO_4_ (Doheny & Ganem, 1980 ▸). [K(18c6)]OAc (OAc = acetate) has been shown to be useful as an acetyl­ation agent for alkyl halides (Liotta *et al.*, 1974 ▸), and over the past few years ‘CECILs’ (crown ether complex cation ionic liquids) such as [K(18c6)]OAc and [K(18c6)]OH gained in importance as basic catalysts for various organic transformations (*e.g.* Song *et al.*, 2011 ▸; Abaszadeh & Seifi, 2015 ▸).

In view of the broad application of 18-crown-6-complexed potassium acetate, [K(18c6)]OAc, it is surprising that the crystal structure of this simple compound has never been determined. In this paper we present the structures of two solvated forms thereof, namely the dihydrate, [K(18c6)]OAc·2 H_2_O (**1**), and the acetic acid hydrate, [K(18c6)]OAc·HOAc·H_2_O (**2**).
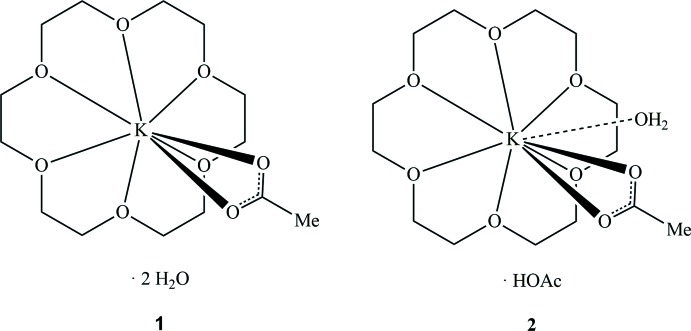


## Structural commentary   

**Figure 1:**
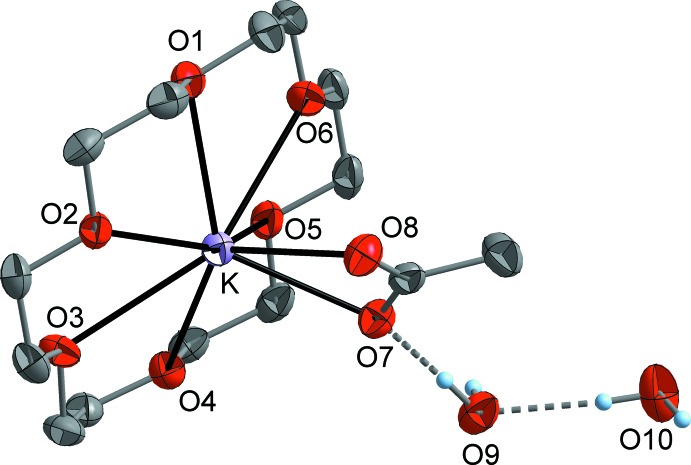
The mol­ecular structure of compound **1**, with displacement ellipsoids drawn at the 50% probability level. C-bound H atoms have been omitted for clarity.

Both title compounds crystallize in the monoclinic space group *P*2_1_/*c* with one formula unit of [K(18c6)]OAc and two solvent mol­ecules in the asymmetric unit. In the dihydrate **1** (Fig. 1 ▸), the potassium atom is coordinated by the crown ether ligand in a slightly unsymmetrical hexa­dentate mode with K—O distances (Table 1 ▸) ranging from 2.8248 (13) to 2.9684 (11) Å and a median value of 2.922 Å. The acetate counter-ion is attached to potassium in a chelating coordination mode where the K—O distances are significantly different with 2.6992 (11) (K—O7) and 2.8861 (11) Å (K—O8). As a result of the additional coordination of the acetate ion, the potassium ion is slightly displaced from the crown ether O_6_ plane. The two water mol­ecules do not coordinate to the potassium ion and inter­act *via* O—H⋯O hydrogen bonds (see *Supra­molecular features* section).

**Figure 2:**
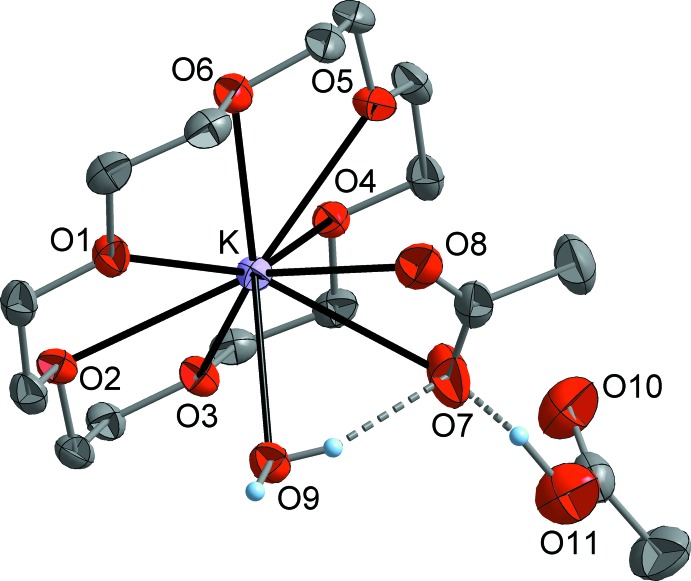
The mol­ecular structure of compound **2**, with dispalcement ellipsoids drawn at the 50% probability level. C-bound H atoms have been omitted for clarity.

By contast, in the acetic acid hydrate **2** (Fig. 2 ▸) the coordination number of the potassium atom is raised to nine by a coordinating water mol­ecule and consequently the K—O bonds (Table 2 ▸) to the acetate ligand are significantly elongated to 2.9562 (16) (K—O8) and 3.0303 (19) Å (K—O7). Moreover, in compound **2** the coordination of the 18c6 ligand is more unsymmetrical than in compound **1** [K—O = 2.7855 (14)–3.0337 (13) Å], but the average K—O distance is virtually identical at 2.920 Å. In general, the geometry of the [K(18c6)]OAc ion pair is not fundamentally influenced by the additional water ligand. Thus, the angle between the KO_2_C(acetate) plane and the O_6_ plane of the 18c6 ligand is similar in both compounds [**1**: 68 (1), **2** 64 (1)°]. The same applies to the displacement of the potassium ion from the O_6_ centroid, which is 0.080 Å in **1** and 0.082 Å in **2**.

The geometry of the [K(18c6)]OAc ion pair in the title compounds fits well with other [K(18c6)]^+^ salts with coordinating anions, *e.g.* the picrate [K(18c6)]O-C_6_H_2_-2,4,6-(NO_2_)_3_ [K—O = 2.862 (4)–2.989 (4) Å, K–centroid(O_6_) 0.0892 (1) Å; Barnes & Collard, 1988 ▸) and the triflate [K(18c6)]OSO_2_CF_3_ [K—O = 2.765 (4)–2.853 (4) Å, K–centroid(O_6_) 0.043 Å; Mandai *et al.*, 2015 ▸). By contrast, in numerous other compounds the potassium ion is situated exactly in the center of the macrocycle and is coordinated symmetrically by the six crown ether O atoms. Since this case resembles the situation in isolated [K(18c6)]^+^ ions, it has been frequently observed in salts with weakly coordinating anions such as thio­cyanate [K—O = 2.768 (1)–2.836 (1) Å, median 2.805 Å] and hexa­fluorido­phosphate [K—O = 2.791 (2)–2.825 (5) Å, median 2.809 Å], where the anions are weakly attached to the potassium ion symmetrically from both sides of the K(18c6)]^+^ cation (Mandai *et al.*, 2015 ▸). Of course, there are also many inter­mediate cases such as the halidomercurates [K(18c6)][Hg(CF_3_)_2_ *X*] (*X* = Br, I; Schulz *et al.*, 2003 ▸). Herein the cation–anion inter­actions are weak in general, but the K⋯*X* inter­action is a little stronger than the K⋯F inter­action on the opposite side of the K(18c6)]^+^ cation and the potassium ion is therefore moved slightly out of the macrocycle [*e.g. X* = I: K—O = 2.768 (6)–2.895 (6) Å, K–centroid(O_6_) = 0.020 Å].

## Supra­molecular features   

**Figure 3:**
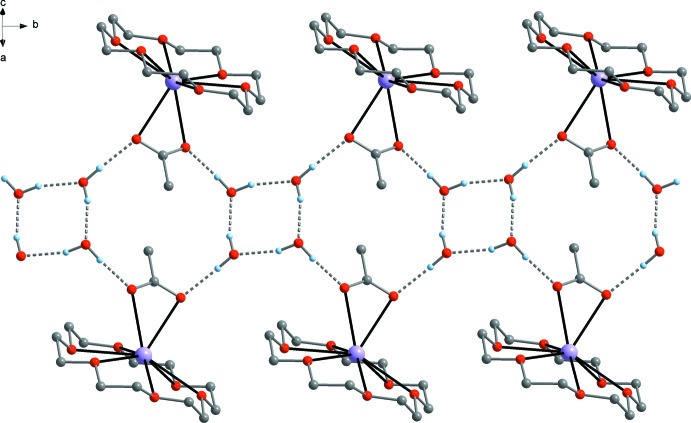
Representation of the polymeric supra­molecular structure of compound **1** linked by O—H⋯O hydrogen bonds. The double-stranded chain extends along the crystallographic *b*-axis direction.

In both title compounds, both acetate oxygen atoms O7 and O8 are involved in hydrogen bonding (Table 3 ▸). In the case of compound **1** (Fig. 3 ▸), two hydrogen atoms of adjacent water mol­ecules donate to the acetate ligand with slightly different O⋯O distances of 2.741 (3) Å (O7⋯O9 including H2) and 2.810 (3) Å (O8⋯O10 including H3). Through the oxygen atom (O9, O10) and the second hydrogen atom (H1, H4) of each water mol­ecule, a cyclic (H_2_O)_4_ moiety is formed with similar O⋯O distances of 2.790 (3) Å (O9⋯O10 including H1) and 2.827 (3) Å (O9⋯O10′ including H4′). By this inter­connection of [K(18c6)(OAc)] moieties and water mol­ecules, a double-stranded polymeric structure is formed. Each chain is characterized by repeating O(H)–H⋯O–C(Me)-O⋯H–O–H units, and through inter­connection of the two chains a ladder-like architecture with alternating C_2_O_8_H_4_ 14-membered rings and O_4_H_4_ eight-membered rings is built. The strength of the hydrogen bonds between the water mol­ecules and acetate ions is similar to those observed in other hydrated metal acetates, *e.g.* [Zn(OAc)_2_(Diap)_2_]·H_2_O [Diap = *cyclo*-C_4_H_10_N_2_C=S; O⋯O = 2.773 (2)–2.814 (1) Å; Beheshti *et al.*, 2007 ▸] and [{Na_2_Cu(OAc)_4_(H_2_O)·H_2_O}_*n*_] [O⋯O = 2.764 (4)–2.944 (8) Å; Li *et al.*, 2010 ▸].

**Figure 4:**
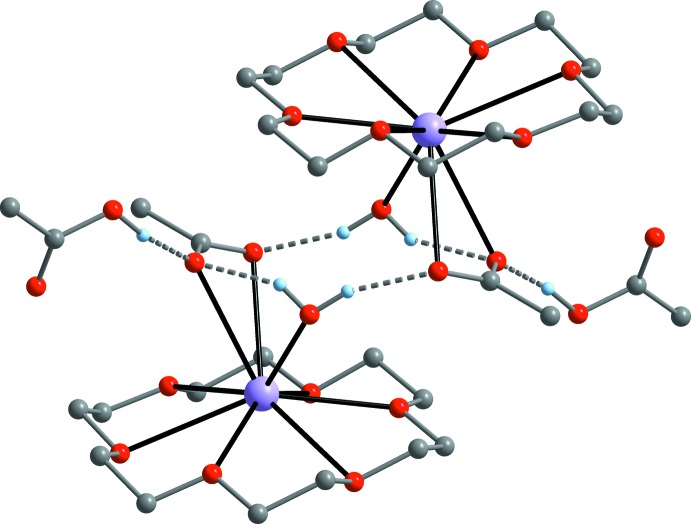
Representation of the dimeric supra­molecular structure of compound **2** arising from O—H⋯O hydrogen bonding.

In the acetic acid hydrate **2** (Fig. 4 ▸), the acetate ligand accepts two O—H⋯O hydrogen bonds (Table 4 ▸) from water mol­ecules with very similar O⋯O distances, an intra­molecular one to the K-coordinating water mol­ecule [O7⋯O9 = 2.785 (7) Å, including H1] and an inter­molecular one to an adjacent [K(18c6)(OAc)(H_2_O)] moiety [O8⋯O9′ = 2.786 (7) Å, including H2′]. In addition, one of the acetate oxygen atoms is attached to the acetic acid mol­ecule with a considerably stronger O—H⋯O bond [O7⋯O11 = 2.513 (6) Å, including H3]. The strength of this hydrogen bond between the acetic acid mol­ecule and acetate ion is comparable with that observed in non-complexed KOAc·HOAc [O⋯O = 2.476 (8) Å; Currie, 1972 ▸] and in NaOAc·HOAc [O⋯O = 2.48 (1) Å; Barrow *et al.*, 1975 ▸]. Neither of the oxygen atoms of the acetic acid moiety in compound **2** (O10, O11) are involved in hydrogen bonding and consequently the supra­molecular structure is simpler than that of compound **1**. Namely, through inter­connection of two [K(18c6)(OAc)(H_2_O)] moieties by the aforementioned H_2_O⋯OAc bridges, a dimeric structure with a centrosymmetric C_2_O_6_H_4_ ring is present.

## Database survey   

For other structurally characterized salts with the [K(18c6)]^+^ cation, see: Neier *et al.* (1995 ▸), Sievert *et al.* (1996 ▸), Schulz *et al.* (2003 ▸), Makarov *et al.* (2005 ▸) and Mandai *et al.* (2015 ▸). For a review of metal complexes with crown ethers, see: Dalley (1978 ▸) and Shono (1994 ▸).

For other structurally characterized hydrates and acetic acid solvates of metal acetates, see: Currie (1972 ▸), Barrow *et al.* (1975 ▸), Beheshti *et al.* (2007 ▸) and Li *et al.* (2010 ▸).

## Synthesis and crystallization   

Single crystals of the title compounds were obtained by slow evaporation of a solution of commercial available potassium acetate in the presence of an equimolar amount of 18-crown-6 in water (**1**) or in diluted acetic acid (**2**).

## Refinement   

Crystal data, data collection and structure refinement details are summarized in Table 5 ▸. All H atoms were fixed geometrically and refined using a riding model with *U*_iso_(H) = 1.2*U*_eq_(C). C—H distances in CH_3_ groups were constrained to 0.98 Å and those in CH_2_ groups to 0.99 Å. Methyl H atoms were allowed to rotate around the C—C vector (AFIX 137 in *SHELXL*). O—H distances within H_2_O mol­ecules were restrained to 0.96 Å (DFIX restraint in *SHELXL*; the s.u. applied was 0.01 Å), while the coordinates of the HOAc hydrogen atom H3 in compound **2** was refined freely.

## Supplementary Material

Crystal structure: contains datablock(s) 1, 2. DOI: 10.1107/S2056989016017436/hb7621sup1.cif

Structure factors: contains datablock(s) 1. DOI: 10.1107/S2056989016017436/hb76211sup2.hkl

Structure factors: contains datablock(s) 2. DOI: 10.1107/S2056989016017436/hb76212sup3.hkl

CCDC references: 1513648, 1513649

Additional supporting information:  crystallographic information; 3D view; checkCIF report

## Figures and Tables

**Table 1:** Selected bond lengths (Å) for **1** [Chem scheme1]

K—O1	2.9684 (11)	K—O5	2.9506 (12)
K—O2	2.8649 (10)	K—O6	2.8248 (13)
K—O3	2.9244 (11)	K—O7	2.6992 (11)
K—O4	2.9380 (11)	K—O8	2.8861 (11)

**Table 2:** Selected bond lengths (Å) for **2** [Chem scheme1]

K—O1	2.7861 (12)	K—O6	2.9435 (13)
K—O2	3.0045 (13)	K—O7	3.0303 (19)
K—O3	2.8510 (12)	K—O8	2.9562 (16)
K—O4	3.0337 (13)	K—O9	2.7855 (14)
K—O5	2.9019 (13)		

**Table 3:** Hydrogen-bond geometry (Å, °) for (1)

*D*—H⋯*A*	*D*—H	H⋯*A*	*D*⋯*A*	*D*—H⋯*A*
O9—H1⋯O10^i^	0.94 (1)	1.88 (1)	2.790 (3)	165 (4)
O9—H2⋯O7^i^	0.93 (1)	1.81 (2)	2.741 (3)	175 (4)
O10—H3⋯O8^ii^	0.93 (1)	1.88 (2)	2.810 (3)	175 (4)
O10—H4⋯O9	0.93 (1)	1.92 (1)	2.827 (3)	166 (4)

Symmetry codes: (i) 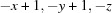; (ii) 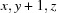.

**Table 4:** Hydrogen-bond geometry (Å, °) for (**2**)

*D*—H⋯*A*	*D*—H	H⋯*A*	*D*⋯*A*	*D*—H⋯*A*
O9—H1⋯O7	0.94 (1)	1.93 (2)	2.785 (7)	151 (9)
O9—H2⋯O8^i^	0.94 (1)	1.89 (2)	2.786 (7)	160 (9)
O11—H3⋯O7	1.08 (5)	1.43 (5)	2.513 (6)	168 (5)

Symmetry code: (i) 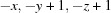.

**Table 5:** Experimental details

	**1**	**2**
Crystal data		
Chemical formula	[K(C_2_H_3_O_2_)(C_12_H_24_O_6_)]·2H_2_O	[K(C_2_H_3_O_2_)(C_12_H_24_O_6_)(H_2_O)]·C_2_H_4_O_2_
*M*_r_	398.49	440.52
Crystal system, space group	Monoclinic, *P*2_1_/*c*	Monoclinic, *P*2_1_/*c*
Temperature (K)	173	200
*a*, *b*, *c* (Å)	11.683 (2), 8.594 (2), 20.083 (4)	11.3233 (1), 8.5450 (1), 23.3869 (3)
β (°)	100.59 (3)	99.053 (1)
*V* (Å^3^)	1982.1 (7)	2234.67 (4)
*Z*	4	4
Radiation type	Mo *K*α	Mo *K*α
μ (mm^−1^)	0.31	0.29
Crystal size (mm)	0.60 × 0.40 × 0.30	0.40 × 0.40 × 0.20

Data collection		
Diffractometer	Bruker SMART CCD	Bruker SMART CCD
Absorption correction	Multi-scan (*SADABS*; Bruker, 2001 ▸)	Multi-scan (*SADABS*; Bruker, 2001 ▸)
*T*_min_, *T*_max_	0.834, 0.912	0.893, 0.945
No. of measured, independent and observed [*I* > 2σ(*I*)] reflections	11876, 4316, 3626	13151, 4846, 3833
*R*_int_	0.025	0.035
(sin θ/λ)_max_ (Å^−1^)	0.639	0.639

Refinement		
*R*[*F* ^2^ > 2σ(*F* ^2^)], *wR*(*F* ^2^), *S*	0.031, 0.080, 1.04	0.041, 0.104, 1.05
No. of reflections	4316	4846
No. of parameters	244	268
No. of restraints	4	2
H-atom treatment	H atoms treated by a mixture of independent and constrained refinement	H atoms treated by a mixture of independent and constrained refinement
Δρ_max_, Δρ_min_ (e Å^−3^)	0.19, −0.25	0.43, −0.31

Computer programs: *SMART* and *SAINT* (Bruker, 2001 ▸), *SHELXS97* (Sheldrick, 2008 ▸), *SHELXL2016* (Sheldrick, 2015 ▸), *DIAMOND* (Brandenburg, 1999 ▸) and *publCIF* (Westrip, 2010 ▸).

## supplementary crystallographic information

### (1) (Acetato-κ2O,O')(1,4,7,10,13,16-hexaoxacyclooctadecane-κ6O)potassium dihydrate Crystal data

**Table:**

[K(C_2_H_3_O_2_)(C_12_H_24_O_6_)]·2H_2_O	*F*(000) = 856
*M**_r_* = 398.49	*D*_x_ = 1.335 Mg m^−^^3^
Monoclinic, *P*2_1_/*c*	Mo *K*α radiation, λ = 0.71073 Å
*a* = 11.683 (2) Å	Cell parameters from 100 reflections
*b* = 8.594 (2) Å	θ = 2.0–27.5°
*c* = 20.083 (4) Å	µ = 0.31 mm^−^^1^
β = 100.59 (3)°	*T* = 173 K
*V* = 1982.1 (7) Å^3^	Block, colorless
*Z* = 4	0.60 × 0.40 × 0.30 mm

### (1) (Acetato-κ2O,O')(1,4,7,10,13,16-hexaoxacyclooctadecane-κ6O)potassium dihydrate Data collection

**Table:**

Bruker SMART CCD diffractometer	3626 reflections with *I* > 2σ(*I*)
Radiation source: fine-focus sealed tube	*R*_int_ = 0.025
ω scans	θ_max_ = 27.0°, θ_min_ = 1.8°
Absorption correction: multi-scan (SADABS; Bruker, 2001)	*h* = −14→14
*T*_min_ = 0.834, *T*_max_ = 0.912	*k* = −10→10
11876 measured reflections	*l* = −25→12
4316 independent reflections	

### (1) (Acetato-κ2O,O')(1,4,7,10,13,16-hexaoxacyclooctadecane-κ6O)potassium dihydrate Refinement

**Table:**

Refinement on *F*^2^	Secondary atom site location: difference Fourier map
Least-squares matrix: full	Hydrogen site location: mixed
*R*[*F*^2^ > 2σ(*F*^2^)] = 0.031	H atoms treated by a mixture of independent and constrained refinement
*wR*(*F*^2^) = 0.080	*w* = 1/[σ^2^(*F*_o_^2^) + (0.0364*P*)^2^ + 0.3159*P*] where *P* = (*F*_o_^2^ + 2*F*_c_^2^)/3
*S* = 1.04	(Δ/σ)_max_ < 0.001
4316 reflections	Δρ_max_ = 0.19 e Å^−^^3^
244 parameters	Δρ_min_ = −0.25 e Å^−^^3^
4 restraints	Extinction correction: SHELXL2016 (Sheldrick, 2015), Fc^*^=kFc[1+0.001xFc^2^λ^3^/sin(2θ)]^-1/4^
Primary atom site location: structure-invariant direct methods	Extinction coefficient: 0.0102 (9)

### (1) (Acetato-κ2O,O')(1,4,7,10,13,16-hexaoxacyclooctadecane-κ6O)potassium dihydrate Special details

**Table:**

Geometry. All esds (except the esd in the dihedral angle between two l.s. planes) are estimated using the full covariance matrix. The cell esds are taken into account individually in the estimation of esds in distances, angles and torsion angles; correlations between esds in cell parameters are only used when they are defined by crystal symmetry. An approximate (isotropic) treatment of cell esds is used for estimating esds involving l.s. planes.

### (1) (Acetato-κ2O,O')(1,4,7,10,13,16-hexaoxacyclooctadecane-κ6O)potassium dihydrate Fractional atomic coordinates and isotropic or equivalent isotropic displacement parameters (Å^2^)

**Table:**

	*x*	*y*	*z*	*U*_iso_*/*U*_eq_	
C1	0.19215 (12)	−0.24843 (16)	0.26697 (7)	0.0406 (3)	
H1A	0.221033	−0.313862	0.307113	0.049*	
H1B	0.230318	−0.283302	0.229444	0.049*	
C2	0.06322 (12)	−0.26499 (16)	0.24703 (7)	0.0382 (3)	
H2A	0.041712	−0.376538	0.244126	0.046*	
H2B	0.024397	−0.215362	0.281429	0.046*	
C3	−0.09719 (11)	−0.19603 (16)	0.16312 (7)	0.0374 (3)	
H3A	−0.133676	−0.141690	0.197352	0.045*	
H3B	−0.124628	−0.305238	0.160118	0.045*	
C4	−0.13129 (12)	−0.11823 (15)	0.09584 (7)	0.0391 (3)	
H4A	−0.091094	−0.168296	0.062154	0.047*	
H4B	−0.216334	−0.127743	0.079787	0.047*	
C5	−0.14346 (12)	0.13078 (17)	0.04410 (7)	0.0403 (3)	
H5A	−0.228583	0.115280	0.030922	0.048*	
H5B	−0.106306	0.096893	0.006017	0.048*	
C6	−0.11728 (11)	0.29849 (16)	0.05959 (7)	0.0377 (3)	
H6A	−0.151622	0.363527	0.020283	0.045*	
H6B	−0.151457	0.330953	0.099006	0.045*	
C7	0.03764 (12)	0.47494 (15)	0.09298 (7)	0.0370 (3)	
H7A	0.004496	0.504928	0.133100	0.044*	
H7B	0.006123	0.546253	0.055350	0.044*	
C8	0.16749 (12)	0.48729 (16)	0.10895 (7)	0.0396 (3)	
H8A	0.201647	0.438366	0.072478	0.048*	
H8B	0.191122	0.598056	0.112413	0.048*	
C9	0.33226 (12)	0.41468 (17)	0.18955 (7)	0.0422 (3)	
H9A	0.359744	0.523840	0.191439	0.051*	
H9B	0.367361	0.358512	0.155230	0.051*	
C10	0.36735 (11)	0.33922 (18)	0.25737 (7)	0.0430 (3)	
H10A	0.452159	0.351775	0.273446	0.052*	
H10B	0.326178	0.389241	0.290618	0.052*	
C11	0.36879 (11)	0.09996 (19)	0.31478 (7)	0.0452 (4)	
H11A	0.323873	0.143600	0.347687	0.054*	
H11B	0.452733	0.114220	0.333181	0.054*	
C12	0.34212 (12)	−0.0685 (2)	0.30425 (8)	0.0483 (4)	
H12A	0.384333	−0.110725	0.269761	0.058*	
H12B	0.368262	−0.125858	0.347112	0.058*	
C14	0.37145 (11)	0.02001 (15)	0.09102 (6)	0.0323 (3)	
C15	0.50091 (13)	0.0405 (2)	0.09116 (9)	0.0514 (4)	
H15A	0.535582	−0.060718	0.084288	0.062*	
H15B	0.538963	0.084059	0.134728	0.062*	
H15C	0.511530	0.111363	0.054547	0.062*	
O1	0.21942 (8)	−0.08938 (11)	0.28225 (5)	0.0363 (2)	
O2	0.02642 (7)	−0.19209 (10)	0.18282 (4)	0.0337 (2)	
O3	−0.09923 (8)	0.04182 (10)	0.10328 (4)	0.0358 (2)	
O4	0.00621 (8)	0.31881 (10)	0.07425 (4)	0.0353 (2)	
O5	0.20816 (8)	0.41003 (11)	0.17174 (5)	0.0371 (2)	
O6	0.33857 (8)	0.17840 (12)	0.25161 (5)	0.0421 (2)	
O7	0.30476 (8)	0.13088 (11)	0.06867 (5)	0.0371 (2)	
O8	0.33793 (9)	−0.10438 (11)	0.11438 (5)	0.0411 (2)	
O9	0.63538 (9)	0.64950 (12)	0.01773 (5)	0.0439 (2)	
H1	0.6336 (18)	0.5550 (15)	−0.0055 (9)	0.073 (6)*	
H2	0.6560 (16)	0.7200 (18)	−0.0134 (8)	0.068 (6)*	
O10	0.40196 (10)	0.64828 (13)	0.03746 (7)	0.0561 (3)	
H3	0.3823 (17)	0.7270 (19)	0.0651 (9)	0.078 (6)*	
H4	0.4786 (9)	0.666 (2)	0.0337 (10)	0.072 (6)*	
K	0.15130 (2)	0.07687 (3)	0.15141 (2)	0.03251 (9)	

### (1) (Acetato-κ2O,O')(1,4,7,10,13,16-hexaoxacyclooctadecane-κ6O)potassium dihydrate Atomic displacement parameters (Å^2^)

**Table:**

	*U*^11^	*U*^22^	*U*^33^	*U*^12^	*U*^13^	*U*^23^
C1	0.0477 (8)	0.0403 (7)	0.0343 (7)	0.0144 (6)	0.0086 (6)	0.0010 (5)
C2	0.0502 (8)	0.0332 (7)	0.0334 (7)	0.0008 (6)	0.0135 (6)	0.0018 (5)
C3	0.0289 (6)	0.0326 (7)	0.0528 (8)	−0.0046 (5)	0.0130 (5)	−0.0003 (6)
C4	0.0320 (6)	0.0335 (7)	0.0490 (8)	−0.0077 (5)	0.0002 (6)	−0.0065 (6)
C5	0.0383 (7)	0.0467 (8)	0.0315 (7)	−0.0040 (6)	−0.0054 (5)	0.0027 (6)
C6	0.0340 (7)	0.0431 (7)	0.0344 (7)	0.0025 (6)	0.0022 (5)	0.0097 (6)
C7	0.0498 (8)	0.0300 (6)	0.0330 (7)	−0.0010 (6)	0.0122 (6)	−0.0020 (5)
C8	0.0503 (8)	0.0352 (7)	0.0367 (7)	−0.0104 (6)	0.0169 (6)	−0.0043 (6)
C9	0.0336 (7)	0.0474 (8)	0.0496 (8)	−0.0148 (6)	0.0180 (6)	−0.0163 (6)
C10	0.0264 (6)	0.0584 (9)	0.0453 (8)	−0.0128 (6)	0.0094 (5)	−0.0213 (7)
C11	0.0249 (6)	0.0722 (10)	0.0360 (7)	0.0049 (6)	−0.0008 (5)	−0.0059 (7)
C12	0.0284 (7)	0.0660 (10)	0.0488 (8)	0.0141 (7)	0.0029 (6)	0.0021 (7)
C14	0.0370 (6)	0.0346 (7)	0.0254 (6)	−0.0023 (5)	0.0057 (5)	−0.0034 (5)
C15	0.0366 (7)	0.0540 (9)	0.0601 (10)	−0.0016 (7)	−0.0002 (6)	0.0185 (7)
O1	0.0293 (4)	0.0430 (5)	0.0365 (5)	0.0083 (4)	0.0056 (4)	0.0000 (4)
O2	0.0297 (4)	0.0368 (5)	0.0358 (5)	−0.0009 (4)	0.0090 (3)	0.0042 (4)
O3	0.0361 (5)	0.0321 (5)	0.0345 (5)	−0.0052 (4)	−0.0056 (4)	−0.0002 (4)
O4	0.0352 (5)	0.0319 (5)	0.0384 (5)	−0.0017 (4)	0.0063 (4)	−0.0023 (4)
O5	0.0333 (5)	0.0428 (5)	0.0369 (5)	−0.0102 (4)	0.0111 (4)	−0.0038 (4)
O6	0.0367 (5)	0.0542 (6)	0.0341 (5)	−0.0079 (4)	0.0035 (4)	−0.0097 (4)
O7	0.0401 (5)	0.0349 (5)	0.0380 (5)	0.0031 (4)	0.0111 (4)	0.0016 (4)
O8	0.0480 (6)	0.0338 (5)	0.0443 (5)	0.0002 (4)	0.0162 (4)	0.0032 (4)
O9	0.0538 (6)	0.0397 (6)	0.0411 (5)	−0.0111 (5)	0.0161 (5)	−0.0043 (4)
O10	0.0452 (6)	0.0414 (6)	0.0854 (9)	−0.0081 (5)	0.0213 (6)	−0.0203 (6)
K	0.03047 (15)	0.03681 (16)	0.03057 (15)	−0.00472 (11)	0.00648 (10)	−0.00161 (10)

### (1) (Acetato-κ2O,O')(1,4,7,10,13,16-hexaoxacyclooctadecane-κ6O)potassium dihydrate Geometric parameters (Å, º)

**Table:**

C1—O1	1.4241 (17)	C9—H9B	0.9900
C1—C2	1.493 (2)	C10—O6	1.4220 (18)
C1—H1A	0.9900	C10—H10A	0.9900
C1—H1B	0.9900	C10—H10B	0.9900
C2—O2	1.4271 (15)	C11—O6	1.4227 (17)
C2—H2A	0.9900	C11—C12	1.488 (2)
C2—H2B	0.9900	C11—H11A	0.9900
C3—O2	1.4259 (15)	C11—H11B	0.9900
C3—C4	1.495 (2)	C12—O1	1.4316 (16)
C3—H3A	0.9900	C12—H12A	0.9900
C3—H3B	0.9900	C12—H12B	0.9900
C4—O3	1.4261 (16)	C14—O8	1.2584 (16)
C4—H4A	0.9900	C14—O7	1.2601 (16)
C4—H4B	0.9900	C14—C15	1.5222 (19)
C5—O3	1.4277 (16)	C14—K	3.0775 (14)
C5—C6	1.494 (2)	C15—H15A	0.9800
C5—H5A	0.9900	C15—H15B	0.9800
C5—H5B	0.9900	C15—H15C	0.9800
C6—O4	1.4292 (15)	K—O1	2.9684 (11)
C6—H6A	0.9900	K—O2	2.8649 (10)
C6—H6B	0.9900	K—O3	2.9244 (11)
C7—O4	1.4234 (16)	K—O4	2.9380 (11)
C7—C8	1.4956 (19)	K—O5	2.9506 (12)
C7—H7A	0.9900	K—O6	2.8248 (13)
C7—H7B	0.9900	K—O7	2.6992 (11)
C8—O5	1.4270 (17)	K—O8	2.8861 (11)
C8—H8A	0.9900	O9—H1	0.935 (9)
C8—H8B	0.9900	O9—H2	0.933 (9)
C9—O5	1.4287 (16)	O10—H3	0.930 (9)
C9—C10	1.496 (2)	O10—H4	0.925 (9)
C9—H9A	0.9900		
			
O1—C1—C2	108.91 (11)	O1—C12—H12A	109.7
O1—C1—H1A	109.9	C11—C12—H12A	109.7
C2—C1—H1A	109.9	O1—C12—H12B	109.7
O1—C1—H1B	109.9	C11—C12—H12B	109.7
C2—C1—H1B	109.9	H12A—C12—H12B	108.2
H1A—C1—H1B	108.3	O8—C14—O7	124.10 (12)
O2—C2—C1	108.79 (11)	O8—C14—C15	118.35 (12)
O2—C2—H2A	109.9	O7—C14—C15	117.53 (12)
C1—C2—H2A	109.9	O8—C14—K	69.40 (7)
O2—C2—H2B	109.9	O7—C14—K	60.89 (7)
C1—C2—H2B	109.9	C15—C14—K	152.01 (10)
H2A—C2—H2B	108.3	C14—C15—H15A	109.5
O2—C3—C4	109.16 (10)	C14—C15—H15B	109.5
O2—C3—H3A	109.8	H15A—C15—H15B	109.5
C4—C3—H3A	109.8	C14—C15—H15C	109.5
O2—C3—H3B	109.8	H15A—C15—H15C	109.5
C4—C3—H3B	109.8	H15B—C15—H15C	109.5
H3A—C3—H3B	108.3	C1—O1—C12	110.99 (10)
O3—C4—C3	108.40 (11)	C1—O1—K	104.96 (7)
O3—C4—H4A	110.0	C12—O1—K	107.66 (8)
C3—C4—H4A	110.0	C3—O2—C2	111.21 (10)
O3—C4—H4B	110.0	C3—O2—K	119.27 (7)
C3—C4—H4B	110.0	C2—O2—K	118.17 (7)
H4A—C4—H4B	108.4	C4—O3—C5	112.42 (10)
O3—C5—C6	108.32 (10)	C4—O3—K	111.20 (7)
O3—C5—H5A	110.0	C5—O3—K	114.02 (8)
C6—C5—H5A	110.0	C7—O4—C6	111.79 (10)
O3—C5—H5B	110.0	C7—O4—K	115.59 (7)
C6—C5—H5B	110.0	C6—O4—K	119.00 (7)
H5A—C5—H5B	108.4	C8—O5—C9	111.47 (10)
O4—C6—C5	108.72 (11)	C8—O5—K	107.42 (7)
O4—C6—H6A	109.9	C9—O5—K	104.72 (8)
C5—C6—H6A	109.9	C10—O6—C11	111.82 (11)
O4—C6—H6B	109.9	C10—O6—K	119.92 (8)
C5—C6—H6B	109.9	C11—O6—K	121.45 (8)
H6A—C6—H6B	108.3	C14—O7—K	95.04 (7)
O4—C7—C8	109.02 (11)	C14—O8—K	86.51 (8)
O4—C7—H7A	109.9	H1—O9—H2	102.4 (17)
C8—C7—H7A	109.9	H3—O10—H4	106.0 (17)
O4—C7—H7B	109.9	O7—K—O6	82.55 (3)
C8—C7—H7B	109.9	O7—K—O2	134.19 (3)
H7A—C7—H7B	108.3	O6—K—O2	116.84 (3)
O5—C8—C7	108.44 (10)	O7—K—O8	46.80 (3)
O5—C8—H8A	110.0	O6—K—O8	80.06 (3)
C7—C8—H8A	110.0	O2—K—O8	93.48 (3)
O5—C8—H8B	110.0	O7—K—O3	123.50 (3)
C7—C8—H8B	110.0	O6—K—O3	149.82 (3)
H8A—C8—H8B	108.4	O2—K—O3	58.02 (3)
O5—C9—C10	108.15 (11)	O8—K—O3	128.09 (3)
O5—C9—H9A	110.1	O7—K—O4	86.43 (3)
C10—C9—H9A	110.1	O6—K—O4	116.96 (3)
O5—C9—H9B	110.1	O2—K—O4	114.79 (3)
C10—C9—H9B	110.1	O8—K—O4	129.49 (3)
H9A—C9—H9B	108.4	O3—K—O4	56.85 (3)
O6—C10—C9	109.03 (11)	O7—K—O5	76.12 (3)
O6—C10—H10A	109.9	O6—K—O5	58.43 (3)
C9—C10—H10A	109.9	O2—K—O5	149.68 (3)
O6—C10—H10B	109.9	O8—K—O5	113.44 (3)
C9—C10—H10B	109.9	O3—K—O5	109.39 (3)
H10A—C10—H10B	108.3	O4—K—O5	58.64 (3)
O6—C11—C12	108.85 (11)	O7—K—O1	121.82 (3)
O6—C11—H11A	109.9	O6—K—O1	58.09 (3)
C12—C11—H11A	109.9	O2—K—O1	58.75 (3)
O6—C11—H11B	109.9	O8—K—O1	82.87 (3)
C12—C11—H11B	109.9	O3—K—O1	109.41 (4)
H11A—C11—H11B	108.3	O4—K—O1	147.39 (3)
O1—C12—C11	109.71 (11)	O5—K—O1	109.13 (3)
			
O1—C1—C2—O2	69.56 (13)	C6—C5—O3—K	−58.71 (12)
O2—C3—C4—O3	−64.30 (14)	C8—C7—O4—C6	178.85 (10)
O3—C5—C6—O4	63.29 (14)	C8—C7—O4—K	38.46 (12)
O4—C7—C8—O5	−71.79 (13)	C5—C6—O4—C7	−176.94 (10)
O5—C9—C10—O6	66.16 (13)	C5—C6—O4—K	−38.05 (12)
O6—C11—C12—O1	−63.66 (15)	C7—C8—O5—C9	178.87 (10)
C2—C1—O1—C12	177.74 (11)	C7—C8—O5—K	64.69 (11)
C2—C1—O1—K	−66.22 (11)	C10—C9—O5—C8	177.00 (11)
C11—C12—O1—C1	175.73 (11)	C10—C9—O5—K	−67.16 (10)
C11—C12—O1—K	61.37 (12)	C9—C10—O6—C11	−179.41 (10)
C4—C3—O2—C2	179.89 (10)	C9—C10—O6—K	−27.92 (13)
C4—C3—O2—K	36.94 (13)	C12—C11—O6—C10	−177.04 (11)
C1—C2—O2—C3	−176.43 (11)	C12—C11—O6—K	31.96 (14)
C1—C2—O2—K	−33.03 (13)	O8—C14—O7—K	30.29 (13)
C3—C4—O3—C5	−171.43 (11)	C15—C14—O7—K	−148.16 (11)
C3—C4—O3—K	59.34 (12)	O7—C14—O8—K	−28.09 (12)
C6—C5—O3—C4	173.54 (11)	C15—C14—O8—K	150.35 (11)

### (1) (Acetato-κ2O,O')(1,4,7,10,13,16-hexaoxacyclooctadecane-κ6O)potassium dihydrate Hydrogen-bond geometry (Å, º)

**Table:**

*D*—H···*A*	*D*—H	H···*A*	*D*···*A*	*D*—H···*A*
O9—H1···O10^i^	0.94 (1)	1.88 (1)	2.790 (3)	165 (4)
O9—H2···O7^i^	0.93 (1)	1.81 (2)	2.741 (3)	175 (4)
O10—H3···O8^ii^	0.93 (1)	1.88 (2)	2.810 (3)	175 (4)
O10—H4···O9	0.93 (1)	1.92 (1)	2.827 (3)	166 (4)

Symmetry codes: (i) −*x*+1, −*y*+1, −*z*; (ii) *x*, *y*+1, *z*.

### (2) (Acetato-κ2O,O')aqua(1,4,7,10,13,16-hexaoxacyclooctadecane-κ6O)potassium acetic acid monosolvate Crystal data

**Table:**

[K(C_2_H_3_O_2_)(C_12_H_24_O_6_)(H_2_O)]·C_2_H_4_O_2_	*F*(000) = 944
*M**_r_* = 440.52	*D*_x_ = 1.309 Mg m^−^^3^
Monoclinic, *P*2_1_/*c*	Mo *K*α radiation, λ = 0.71073 Å
*a* = 11.3233 (1) Å	Cell parameters from 100 reflections
*b* = 8.5450 (1) Å	θ = 2.0–27.5°
*c* = 23.3869 (3) Å	µ = 0.29 mm^−^^1^
β = 99.053 (1)°	*T* = 200 K
*V* = 2234.67 (4) Å^3^	Block, colorless
*Z* = 4	0.40 × 0.40 × 0.20 mm

### (2) (Acetato-κ2O,O')aqua(1,4,7,10,13,16-hexaoxacyclooctadecane-κ6O)potassium acetic acid monosolvate Data collection

**Table:**

Bruker SMART CCD diffractometer	3833 reflections with *I* > 2σ(*I*)
Radiation source: fine-focus sealed tube	*R*_int_ = 0.035
ω scans	θ_max_ = 27.0°, θ_min_ = 1.8°
Absorption correction: multi-scan (SADABS; Bruker, 2001)	*h* = −14→14
*T*_min_ = 0.893, *T*_max_ = 0.945	*k* = −10→10
13151 measured reflections	*l* = −29→19
4846 independent reflections	

### (2) (Acetato-κ2O,O')aqua(1,4,7,10,13,16-hexaoxacyclooctadecane-κ6O)potassium acetic acid monosolvate Refinement

**Table:**

Refinement on *F*^2^	Secondary atom site location: difference Fourier map
Least-squares matrix: full	Hydrogen site location: mixed
*R*[*F*^2^ > 2σ(*F*^2^)] = 0.041	H atoms treated by a mixture of independent and constrained refinement
*wR*(*F*^2^) = 0.104	*w* = 1/[σ^2^(*F*_o_^2^) + (0.0411*P*)^2^ + 0.8233*P*] where *P* = (*F*_o_^2^ + 2*F*_c_^2^)/3
*S* = 1.05	(Δ/σ)_max_ = 0.001
4846 reflections	Δρ_max_ = 0.43 e Å^−^^3^
268 parameters	Δρ_min_ = −0.31 e Å^−^^3^
2 restraints	Extinction correction: SHELXL2016 (Sheldrick, 2015), Fc^*^=kFc[1+0.001xFc^2^λ^3^/sin(2θ)]^-1/4^
Primary atom site location: structure-invariant direct methods	Extinction coefficient: 0.0064 (8)

### (2) (Acetato-κ2O,O')aqua(1,4,7,10,13,16-hexaoxacyclooctadecane-κ6O)potassium acetic acid monosolvate Special details

**Table:**

Geometry. All esds (except the esd in the dihedral angle between two l.s. planes) are estimated using the full covariance matrix. The cell esds are taken into account individually in the estimation of esds in distances, angles and torsion angles; correlations between esds in cell parameters are only used when they are defined by crystal symmetry. An approximate (isotropic) treatment of cell esds is used for estimating esds involving l.s. planes.

### (2) (Acetato-κ2O,O')aqua(1,4,7,10,13,16-hexaoxacyclooctadecane-κ6O)potassium acetic acid monosolvate Fractional atomic coordinates and isotropic or equivalent isotropic displacement parameters (Å^2^)

**Table:**

	*x*	*y*	*z*	*U*_iso_*/*U*_eq_	
C1	−0.26514 (15)	0.3641 (2)	0.59639 (8)	0.0344 (4)	
H1A	−0.295602	0.316810	0.629859	0.041*	
H1B	−0.325899	0.348656	0.561493	0.041*	
C2	−0.24318 (16)	0.5356 (2)	0.60680 (8)	0.0339 (4)	
H2A	−0.211614	0.582388	0.573534	0.041*	
H2B	−0.319274	0.588713	0.610600	0.041*	
C3	−0.12935 (17)	0.7194 (2)	0.66672 (8)	0.0326 (4)	
H3A	−0.202139	0.780529	0.670253	0.039*	
H3B	−0.095777	0.759557	0.632950	0.039*	
C4	−0.03993 (17)	0.7378 (2)	0.72029 (8)	0.0322 (4)	
H4A	−0.027326	0.850197	0.729470	0.039*	
H4B	−0.069596	0.686669	0.753275	0.039*	
C5	0.15966 (17)	0.6830 (2)	0.76087 (8)	0.0318 (4)	
H5A	0.136112	0.624324	0.793796	0.038*	
H5B	0.169625	0.794519	0.772089	0.038*	
C6	0.27450 (17)	0.6196 (2)	0.74649 (9)	0.0347 (4)	
H6A	0.292850	0.668968	0.710610	0.042*	
H6B	0.340818	0.642839	0.778267	0.042*	
C7	0.36809 (16)	0.3840 (2)	0.72475 (9)	0.0357 (4)	
H7A	0.436684	0.406551	0.755438	0.043*	
H7B	0.386265	0.426119	0.687675	0.043*	
C8	0.34709 (17)	0.2111 (2)	0.71987 (9)	0.0374 (5)	
H8A	0.421645	0.157251	0.713968	0.045*	
H8B	0.322839	0.170756	0.755975	0.045*	
C9	0.22477 (18)	0.0196 (2)	0.66723 (9)	0.0373 (5)	
H9A	0.188685	−0.013663	0.701176	0.045*	
H9B	0.297603	−0.043805	0.666202	0.045*	
C10	0.13805 (17)	−0.0062 (2)	0.61311 (9)	0.0370 (5)	
H10A	0.170412	0.037599	0.579565	0.044*	
H10B	0.124689	−0.119710	0.606545	0.044*	
C11	−0.05449 (18)	0.0615 (2)	0.56565 (8)	0.0347 (4)	
H11A	−0.065285	−0.048774	0.552956	0.042*	
H11B	−0.022018	0.120701	0.535208	0.042*	
C12	−0.17179 (17)	0.1291 (2)	0.57418 (8)	0.0341 (4)	
H12A	−0.230412	0.117291	0.538289	0.041*	
H12B	−0.202776	0.072861	0.605745	0.041*	
C13	0.26513 (17)	0.4042 (3)	0.54918 (9)	0.0395 (5)	
C14	0.3933 (2)	0.3558 (4)	0.54951 (12)	0.0628 (7)	
H14A	0.441672	0.448409	0.544505	0.075*	
H14B	0.397490	0.282164	0.517759	0.075*	
H14C	0.424067	0.305288	0.586489	0.075*	
C15	0.46601 (19)	0.7951 (3)	0.60242 (11)	0.0458 (5)	
C16	0.5436 (3)	0.9332 (3)	0.59606 (15)	0.0734 (8)	
H16A	0.588137	0.915124	0.563968	0.088*	
H16B	0.599944	0.948428	0.631968	0.088*	
H16C	0.493720	1.026816	0.588064	0.088*	
O1	−0.15612 (11)	0.29088 (15)	0.58855 (6)	0.0329 (3)	
O2	−0.15927 (11)	0.55729 (14)	0.65841 (5)	0.0299 (3)	
O3	0.06996 (11)	0.66769 (15)	0.71136 (5)	0.0298 (3)	
O4	0.26219 (11)	0.45464 (14)	0.73867 (6)	0.0311 (3)	
O5	0.25553 (11)	0.18135 (14)	0.67223 (5)	0.0322 (3)	
O6	0.02708 (11)	0.06945 (14)	0.61872 (5)	0.0319 (3)	
O7	0.24709 (14)	0.5417 (2)	0.56419 (9)	0.0654 (5)	
O8	0.18335 (13)	0.30957 (19)	0.53554 (6)	0.0459 (4)	
O9	0.01027 (12)	0.64125 (16)	0.55199 (6)	0.0339 (3)	
H2	−0.0421 (19)	0.648 (3)	0.5168 (7)	0.066 (8)*	
H1	0.0852 (12)	0.616 (3)	0.5426 (10)	0.054 (7)*	
O10	0.47534 (17)	0.7214 (2)	0.64660 (8)	0.0707 (5)	
O11	0.39035 (17)	0.7625 (3)	0.55675 (8)	0.0718 (6)	
H3	0.337 (4)	0.662 (6)	0.564 (2)	0.165 (18)*	
K	0.06834 (3)	0.40782 (4)	0.63478 (2)	0.02625 (12)	

### (2) (Acetato-κ2O,O')aqua(1,4,7,10,13,16-hexaoxacyclooctadecane-κ6O)potassium acetic acid monosolvate Atomic displacement parameters (Å^2^)

**Table:**

	*U*^11^	*U*^22^	*U*^33^	*U*^12^	*U*^13^	*U*^23^
C1	0.0225 (9)	0.0459 (12)	0.0334 (10)	−0.0006 (8)	−0.0003 (7)	0.0009 (8)
C2	0.0258 (9)	0.0432 (11)	0.0312 (10)	0.0080 (8)	−0.0002 (7)	0.0040 (8)
C3	0.0370 (10)	0.0278 (10)	0.0342 (10)	0.0096 (8)	0.0097 (8)	0.0035 (8)
C4	0.0393 (10)	0.0265 (9)	0.0328 (10)	0.0053 (8)	0.0114 (8)	−0.0041 (8)
C5	0.0433 (11)	0.0251 (9)	0.0255 (9)	−0.0039 (8)	0.0006 (8)	−0.0026 (7)
C6	0.0352 (10)	0.0311 (10)	0.0357 (10)	−0.0073 (8)	−0.0013 (8)	0.0003 (8)
C7	0.0228 (9)	0.0441 (12)	0.0385 (11)	0.0011 (8)	−0.0003 (8)	−0.0001 (9)
C8	0.0290 (10)	0.0416 (11)	0.0399 (11)	0.0117 (8)	0.0002 (8)	0.0031 (9)
C9	0.0380 (11)	0.0239 (10)	0.0516 (12)	0.0078 (8)	0.0118 (9)	0.0052 (8)
C10	0.0394 (11)	0.0233 (9)	0.0515 (12)	0.0009 (8)	0.0170 (9)	−0.0083 (8)
C11	0.0495 (12)	0.0283 (10)	0.0270 (9)	−0.0070 (8)	0.0083 (8)	−0.0059 (7)
C12	0.0380 (10)	0.0328 (10)	0.0295 (9)	−0.0111 (8)	−0.0010 (8)	−0.0044 (8)
C13	0.0294 (10)	0.0581 (14)	0.0307 (10)	0.0042 (10)	0.0040 (8)	0.0103 (9)
C14	0.0367 (12)	0.083 (2)	0.0697 (17)	0.0152 (12)	0.0132 (12)	0.0046 (15)
C15	0.0327 (11)	0.0464 (13)	0.0581 (14)	0.0032 (9)	0.0066 (10)	−0.0108 (11)
C16	0.0665 (18)	0.0566 (17)	0.094 (2)	−0.0209 (14)	0.0030 (16)	0.0084 (15)
O1	0.0250 (6)	0.0326 (7)	0.0400 (7)	−0.0027 (5)	0.0022 (5)	−0.0051 (6)
O2	0.0319 (7)	0.0295 (7)	0.0273 (6)	0.0038 (5)	0.0014 (5)	0.0018 (5)
O3	0.0332 (7)	0.0316 (7)	0.0241 (6)	0.0051 (5)	0.0027 (5)	−0.0048 (5)
O4	0.0275 (6)	0.0265 (7)	0.0391 (7)	−0.0013 (5)	0.0046 (5)	0.0015 (5)
O5	0.0347 (7)	0.0250 (7)	0.0352 (7)	0.0033 (5)	0.0002 (6)	0.0024 (5)
O6	0.0352 (7)	0.0286 (7)	0.0329 (7)	0.0006 (5)	0.0085 (6)	−0.0048 (5)
O7	0.0289 (8)	0.0635 (12)	0.1036 (15)	−0.0045 (8)	0.0099 (9)	−0.0225 (11)
O8	0.0396 (8)	0.0536 (9)	0.0422 (8)	−0.0021 (7)	−0.0002 (7)	0.0117 (7)
O9	0.0345 (7)	0.0357 (7)	0.0314 (7)	0.0034 (6)	0.0043 (6)	0.0001 (6)
O10	0.0710 (12)	0.0823 (14)	0.0568 (11)	−0.0218 (10)	0.0037 (9)	0.0083 (10)
O11	0.0637 (12)	0.0804 (14)	0.0629 (12)	−0.0156 (10)	−0.0156 (10)	0.0053 (10)
K	0.02579 (19)	0.0254 (2)	0.0272 (2)	0.00122 (15)	0.00280 (14)	0.00003 (15)

### (2) (Acetato-κ2O,O')aqua(1,4,7,10,13,16-hexaoxacyclooctadecane-κ6O)potassium acetic acid monosolvate Geometric parameters (Å, º)

**Table:**

C1—O1	1.421 (2)	C10—H10B	0.9900
C1—C2	1.500 (3)	C11—O6	1.427 (2)
C1—H1A	0.9900	C11—C12	1.490 (3)
C1—H1B	0.9900	C11—H11A	0.9900
C2—O2	1.426 (2)	C11—H11B	0.9900
C2—H2A	0.9900	C12—O1	1.427 (2)
C2—H2B	0.9900	C12—H12A	0.9900
C3—O2	1.432 (2)	C12—H12B	0.9900
C3—C4	1.490 (3)	C13—O8	1.233 (3)
C3—H3A	0.9900	C13—O7	1.252 (3)
C3—H3B	0.9900	C13—C14	1.508 (3)
C4—O3	1.426 (2)	C13—K	3.221 (2)
C4—H4A	0.9900	C14—H14A	0.9800
C4—H4B	0.9900	C14—H14B	0.9800
C5—O3	1.421 (2)	C14—H14C	0.9800
C5—C6	1.495 (3)	C15—O10	1.201 (3)
C5—H5A	0.9900	C15—O11	1.290 (3)
C5—H5B	0.9900	C15—C16	1.493 (3)
C6—O4	1.425 (2)	C16—H16A	0.9800
C6—H6A	0.9900	C16—H16B	0.9800
C6—H6B	0.9900	C16—H16C	0.9800
C7—O4	1.425 (2)	K—O1	2.7861 (12)
C7—C8	1.498 (3)	K—O2	3.0045 (13)
C7—H7A	0.9900	K—O3	2.8510 (12)
C7—H7B	0.9900	K—O4	3.0337 (13)
C8—O5	1.420 (2)	K—O5	2.9019 (13)
C8—H8A	0.9900	K—O6	2.9435 (13)
C8—H8B	0.9900	K—O7	3.0303 (19)
C9—O5	1.426 (2)	K—O8	2.9562 (16)
C9—C10	1.491 (3)	K—O9	2.7855 (14)
C9—H9A	0.9900	O9—H2	0.937 (10)
C9—H9B	0.9900	O9—H1	0.935 (10)
C10—O6	1.437 (2)	O11—H3	1.08 (5)
C10—H10A	0.9900		
			
O1—C1—C2	109.02 (15)	C13—C14—H14A	109.5
O1—C1—H1A	109.9	C13—C14—H14B	109.5
C2—C1—H1A	109.9	H14A—C14—H14B	109.5
O1—C1—H1B	109.9	C13—C14—H14C	109.5
C2—C1—H1B	109.9	H14A—C14—H14C	109.5
H1A—C1—H1B	108.3	H14B—C14—H14C	109.5
O2—C2—C1	109.61 (15)	O10—C15—O11	123.8 (2)
O2—C2—H2A	109.7	O10—C15—C16	121.8 (2)
C1—C2—H2A	109.7	O11—C15—C16	114.4 (2)
O2—C2—H2B	109.7	C15—C16—H16A	109.5
C1—C2—H2B	109.7	C15—C16—H16B	109.5
H2A—C2—H2B	108.2	H16A—C16—H16B	109.5
O2—C3—C4	109.30 (14)	C15—C16—H16C	109.5
O2—C3—H3A	109.8	H16A—C16—H16C	109.5
C4—C3—H3A	109.8	H16B—C16—H16C	109.5
O2—C3—H3B	109.8	C1—O1—C12	112.24 (14)
C4—C3—H3B	109.8	C1—O1—K	123.36 (10)
H3A—C3—H3B	108.3	C12—O1—K	120.72 (10)
O3—C4—C3	109.03 (14)	C2—O2—C3	110.62 (14)
O3—C4—H4A	109.9	C2—O2—K	105.41 (10)
C3—C4—H4A	109.9	C3—O2—K	104.17 (10)
O3—C4—H4B	109.9	C5—O3—C4	111.74 (13)
C3—C4—H4B	109.9	C5—O3—K	121.11 (10)
H4A—C4—H4B	108.3	C4—O3—K	119.97 (10)
O3—C5—C6	108.58 (14)	C7—O4—C6	112.30 (14)
O3—C5—H5A	110.0	C7—O4—K	106.86 (10)
C6—C5—H5A	110.0	C6—O4—K	106.08 (10)
O3—C5—H5B	110.0	C8—O5—C9	112.12 (14)
C6—C5—H5B	110.0	C8—O5—K	121.71 (10)
H5A—C5—H5B	108.4	C9—O5—K	117.67 (10)
O4—C6—C5	108.44 (15)	C11—O6—C10	110.78 (14)
O4—C6—H6A	110.0	C11—O6—K	103.03 (10)
C5—C6—H6A	110.0	C10—O6—K	109.26 (10)
O4—C6—H6B	110.0	C13—O7—K	87.18 (13)
C5—C6—H6B	110.0	C13—O7—H3	121.1 (19)
H6A—C6—H6B	108.4	K—O7—H3	143.3 (19)
O4—C7—C8	107.82 (15)	C13—O8—K	90.94 (13)
O4—C7—H7A	110.1	K—O9—H2	134.4 (17)
C8—C7—H7A	110.1	K—O9—H1	82.7 (15)
O4—C7—H7B	110.1	H2—O9—H1	106 (2)
C8—C7—H7B	110.1	C15—O11—H3	111 (3)
H7A—C7—H7B	108.5	O9—K—O1	83.45 (4)
O5—C8—C7	108.89 (15)	O9—K—O3	81.74 (4)
O5—C8—H8A	109.9	O1—K—O3	116.00 (4)
C7—C8—H8A	109.9	O9—K—O5	140.23 (4)
O5—C8—H8B	109.9	O1—K—O5	117.15 (4)
C7—C8—H8B	109.9	O3—K—O5	113.43 (4)
H8A—C8—H8B	108.3	O9—K—O6	126.82 (4)
O5—C9—C10	109.42 (15)	O1—K—O6	58.76 (4)
O5—C9—H9A	109.8	O3—K—O6	146.14 (4)
C10—C9—H9A	109.8	O5—K—O6	58.48 (4)
O5—C9—H9B	109.8	O9—K—O8	75.08 (4)
C10—C9—H9B	109.8	O1—K—O8	94.65 (4)
H9A—C9—H9B	108.2	O3—K—O8	138.97 (4)
O6—C10—C9	108.94 (15)	O5—K—O8	69.91 (4)
O6—C10—H10A	109.9	O6—K—O8	72.63 (4)
C9—C10—H10A	109.9	O9—K—O2	73.02 (4)
O6—C10—H10B	109.9	O1—K—O2	57.81 (4)
C9—C10—H10B	109.9	O3—K—O2	58.28 (3)
H10A—C10—H10B	108.3	O5—K—O2	146.61 (4)
O6—C11—C12	109.49 (14)	O6—K—O2	108.51 (4)
O6—C11—H11A	109.8	O8—K—O2	139.62 (4)
C12—C11—H11A	109.8	O9—K—O7	57.03 (5)
O6—C11—H11B	109.8	O1—K—O7	124.80 (5)
C12—C11—H11B	109.8	O3—K—O7	96.28 (4)
H11A—C11—H11B	108.2	O5—K—O7	84.11 (4)
O1—C12—C11	109.04 (15)	O6—K—O7	114.05 (4)
O1—C12—H12A	109.9	O8—K—O7	42.69 (5)
C11—C12—H12A	109.9	O2—K—O7	127.27 (4)
O1—C12—H12B	109.9	O9—K—O4	121.61 (4)
C11—C12—H12B	109.9	O1—K—O4	149.21 (4)
H12A—C12—H12B	108.3	O3—K—O4	57.44 (3)
O8—C13—O7	122.60 (19)	O5—K—O4	56.23 (3)
O8—C13—C14	120.3 (2)	O6—K—O4	108.18 (4)
O7—C13—C14	117.1 (2)	O8—K—O4	108.28 (4)
O8—C13—K	66.57 (11)	O2—K—O4	109.28 (4)
O7—C13—K	69.97 (12)	O7—K—O4	85.72 (4)
C14—C13—K	139.36 (15)		
			
O1—C1—C2—O2	−61.89 (19)	C3—C4—O3—K	−29.99 (18)
O2—C3—C4—O3	67.56 (18)	C8—C7—O4—C6	177.77 (15)
O3—C5—C6—O4	−67.82 (18)	C8—C7—O4—K	−66.31 (15)
O4—C7—C8—O5	65.00 (19)	C5—C6—O4—C7	−179.99 (15)
O5—C9—C10—O6	−67.21 (19)	C5—C6—O4—K	63.62 (15)
O6—C11—C12—O1	63.41 (19)	C7—C8—O5—C9	−175.98 (15)
C2—C1—O1—C12	−175.70 (15)	C7—C8—O5—K	−28.8 (2)
C2—C1—O1—K	25.89 (19)	C10—C9—O5—C8	−174.05 (15)
C11—C12—O1—C1	178.99 (15)	C10—C9—O5—K	37.31 (18)
C11—C12—O1—K	−21.96 (18)	C12—C11—O6—C10	175.38 (15)
C1—C2—O2—C3	175.23 (15)	C12—C11—O6—K	−67.88 (14)
C1—C2—O2—K	63.21 (15)	C9—C10—O6—C11	174.01 (15)
C4—C3—O2—C2	−178.92 (14)	C9—C10—O6—K	61.18 (16)
C4—C3—O2—K	−66.11 (14)	O8—C13—O7—K	42.6 (2)
C6—C5—O3—C4	−175.13 (15)	C14—C13—O7—K	−136.11 (18)
C6—C5—O3—K	34.54 (18)	O7—C13—O8—K	−43.9 (2)
C3—C4—O3—C5	179.29 (14)	C14—C13—O8—K	134.79 (19)

### (2) (Acetato-κ2O,O')aqua(1,4,7,10,13,16-hexaoxacyclooctadecane-κ6O)potassium acetic acid monosolvate Hydrogen-bond geometry (Å, º)

**Table:**

*D*—H···*A*	*D*—H	H···*A*	*D*···*A*	*D*—H···*A*
O9—H1···O7	0.94 (1)	1.93 (2)	2.785 (7)	151 (9)
O9—H2···O8^i^	0.94 (1)	1.89 (2)	2.786 (7)	160 (9)
O11—H3···O7	1.08 (5)	1.43 (5)	2.513 (6)	168 (5)

Symmetry code: (i) −*x*, −*y*+1, −*z*+1.
